# The impact of pandemic disruptions on clinical skills learning for pre-clinical medical students: implications for future educational designs

**DOI:** 10.1186/s12909-023-04351-9

**Published:** 2023-05-23

**Authors:** Shannon Saad, Cassandra Richmond, Dane King, Caelyn Jones, Bunmi Malau-Aduli

**Affiliations:** 1 School of Medicine, Notre Dame University, Sydney, NSW Australia; 2 College of Medicine and Dentistry, James Cook University, Townsville, QLD Australia; 3 School of Medicine and Public Health, University of Newcastle, Newcastle, Australia

**Keywords:** Pandemic, Clinical skills, Transition, Clinical placement, Medical students

## Abstract

**Background:**

Pandemic disruptions to medical education worldwide resulted in rapid adaptations to clinical skills learning. These adaptations included moving most teaching to the online environment, decreasing the accepted “hands-on” methods of teaching and learning. While studies have shown significant impacts on student confidence in skills acquisition, there is a paucity of assessment outcome studies which would contribute a valuable perspective on whether measurable deficits were incurred. Here, a preclinical (Year 2) cohort was investigated for clinical skills learning impacts that could influence their transition to hospital-based placements.

**Methods:**

A sequential mixed methods approach was used on the Year 2 Medicine cohort, including: focus group discussions with thematic analysis; a survey derived from the themes observed; and a cohort comparison of the clinical skills examination results of the disrupted Year 2 cohort, compared to pre-pandemic cohorts.

**Results:**

Students reported experiencing benefits and disadvantages of the shift to online learning, including a decrease in confidence in their skills acquisition. End of year summative clinical assessments showed non-inferior outcomes when compared to previous cohorts for the majority of clinical skills. However, for procedural skills (venepuncture) the disrupted cohort had significantly lower scores compared to a pre-pandemic cohort.

**Conclusions:**

Rapid innovation during the COVID-19 pandemic provided the opportunity to compare online asynchronous hybrid clinical skills learning with the usual practice of face-to-face synchronous experiential learning. In this study, students’ reported perceptions and assessment performance data indicate that careful selection of skills suitable for online teaching, supported by timetabled “hands-on” sessions and ample practice opportunities, is likely to provide non-inferior outcomes for clinical skills learning in students about to transition to clinical placements. The findings can be used to inform clinical skills curriculum designs that incorporate the virtual environment, and assist with future-proofing skills teaching in the case of further catastrophic disruptions.

**Supplementary Information:**

The online version contains supplementary material available at 10.1186/s12909-023-04351-9.

## Background

At the heart of medical education delivery lies the consolidation of skills, knowledge and attitudes necessary for optimal patient-centred care [1]. A blended approach of theoretical and practical teaching is central to this pedagogy, whereby students are encouraged to develop a medical knowledge base and acquire ‘hands on’ clinical skills whilst developing professionally through role-modelling and reflective practice [2, 3]. At the end of each given year level, medical students must demonstrate skills to a minimum standard for course progression. For junior medical students preparing to transition from pre-clinical to clinical training, the attainment of basic clinical competence is a requirement prior to their regular engagement with real patients during hospital placements.

Face-to-face clinical skills training is traditionally considered integral to any medical program as this provides ‘hands on’ opportunities for developing profession-specific skills, such as history-taking, effective communication, and performing physical examinations and basic procedural skills [4]. For junior medical students, clinical skills are usually learned during simulation-based tutorials, where students are encouraged to practise skills utilising role-playing patients (who are often peers or volunteers). In this setting, students receive feedback from the medical facilitator (and fellow colleagues) in a safe (classroom) environment, without the direct risk of harm to patients [5].

However, the usual pathways for clinical learning in medical education have been vastly disrupted at many institutions since the advent of the COVID-19 global crisis [6]. In response to physical distancing regulations and repeat lockdown periods, many medical schools have responded by shifting their usual face-to-face clinical skills training approach to a remote learning format. The delivery of this adapted teaching approach has constituted a seismic shift in how medical students traditionally receive their clinical skills education [7]. The immediate concern in shifting to distanced clinical skills training (even for transient periods) is that this results in reduced opportunities for clinical skills practise, especially of physical examinations and basic procedural skills [8]. The potential to negatively impact skills acquisition is particularly worrisome for those students preparing to transition to clinical placements as ‘catch-up’ opportunities for missed skills in a pre-clinical learning environment is generally less readily available once progression to clinical placements has occurred.

During peak pandemic conditions, Australia adopted a “COVID zero” stance whilst vaccination rates were below 70% of the population above 12 years. This approach resulted in multiple state-based lockdown periods during 2020 and 2021. Given the importance of hands-on skills practise in developing basic clinical competence prior to the transition to clinical placements, it is significant to understand the educational impact of pandemic disruptions for pre-clinical students preparing for the shift to clinical training. Evaluating the implications and possible solutions will be particularly relevant in circumstances where the pandemic continues despite the worldwide vaccination drive, and where ongoing local outbreaks continue to result in transient lockdown periods and concomitant shifts to online clinical skills teaching.

To understand the impact of disruptions to clinical skills teaching for students preparing to transition to their clinical placements, the perceptions of Year 2 medical students at the University of Notre Dame, Australia, School of Medicine Sydney (SoMS) were explored in relation to their self-reported learning of clinical skills during 2020. In addition, the 2020 Year 2 Observed Structured Clinical Examination (OSCE) data was compared with the data from the same clinical stations at the (pre-pandemic) 2018 and 2019 OSCE to elucidate whether there was a difference in clinical performances between the COVID-impacted students and those where clinical skills teaching was not disrupted by a pandemic. These findings provide information about the impact of clinical skills teaching disruptions on skill acquisition during pandemic conditions and may provide insights to guide future pedagogical approaches in the context of an ongoing pandemic environment.

## Methods

### Study aims

This study aimed to investigate the perceptions of Year 2 SoMS students regarding their self-reported clinical skills learning during a pandemic-disrupted year (phases 1 and 2a); and compare the overall clinical performance of this cohort at the end-of-year summative OSCE with the performances at the same stations in 2018 and 2019 (phase 2b) to elucidate whether there was a significant difference between the clinical performances (as a marker of skill acquisition) of COVID-impacted students with pre-COVID students.

### Study context

This study was conducted at the University of Notre Dame, School of Medicine, Sydney, a 4-year graduate entry program based in NSW, Australia. Eligible students included all medical students enrolled in Year 2 during 2020. The clinical skills learning experiences of these particular students were examined as this cohort represented the group preparing to shift from pre-clinical training to clinical placements the following year. Further, as these students had experienced undisrupted clinical skills teaching during 2019 (when they were in Year 1), this was a cohort that had experienced both undisrupted and disrupted modalities of clinical skills training during their pre-clinical years (see Table 1 for summary).

**Table 1:** Summary of clinical skills teaching approaches before, during, and after the adaptations in 2020

**Prior to the pandemic; the traditional approach to clinical skills teaching at SoMS**	• Delivered face-to-face as a single, 4-h clinical tutorial each week• Small groups, consisting of 8–9 students, facilitated by a medical facilitator• Case-based simulation with the involvement of a VSP• Students learn history-taking, physical examination and procedural skills
**Remote learning during lockdown**	• Synchronised distanced teaching, with priority placed on learning history-taking and clinical reasoning skills (e.g., investigations planning and interpretation) • Reduced focus on learning physical examination and procedural skills • Shift from hands on practise of skills with involvement of VSP to observing clinical skills demonstration videos and theoretical discussions about clinical techniques
**Return to face-to-face clinical skills teaching following remote learning**	• Return to scheduled clinical skills tutorials on campus • Intensive hands-on practise of physical examinations and procedural skills • Additional, self-directed clinical skills practise on campus through book-in system

In a typical, non-pandemic year, Year 2 clinical skills teaching is delivered face-to-face, in the form of 4-hourly clinical tutorials each week. These are run in small groups (of 8–9 students), involving case-based simulation with the inclusion of volunteer simulated patients (VSPs). During these clinical tutorials, students are encouraged to practise history-taking, physical examinations and procedural skills with the guidance of a medical facilitator. However, during the lockdown period in NSW between March and June 2020, Year 2 face-to-face clinical skills teaching was promptly halted, and online clinical skills teaching commenced. This transient adaptation to synchronised distanced education involved prioritising the practise of history-taking and clinical reasoning skills, while a lesser focus was placed on online learning of physical examination and procedural skills. In lieu of hands on practise during distanced learning, students were encouraged to observe clinical skills video demonstrations, and to engage in theoretical discussions about clinical techniques. The return to face-to-face clinical skills training in the second half of 2020 meant that the SoMS Year 2 students could attend their scheduled clinical skills tutorials for the remainder of the academic year. These consisted of intensive clinical skills catch-up sessions, focussing mainly on physical examination and procedural skills practise. Year 2 students had additional opportunities to book clinical rooms for self-directed clinical skills practise. However, this opportunity was restricted by ongoing physical distancing mandates and limited room capacities on campus.

### Study design

**Fig. 1:**
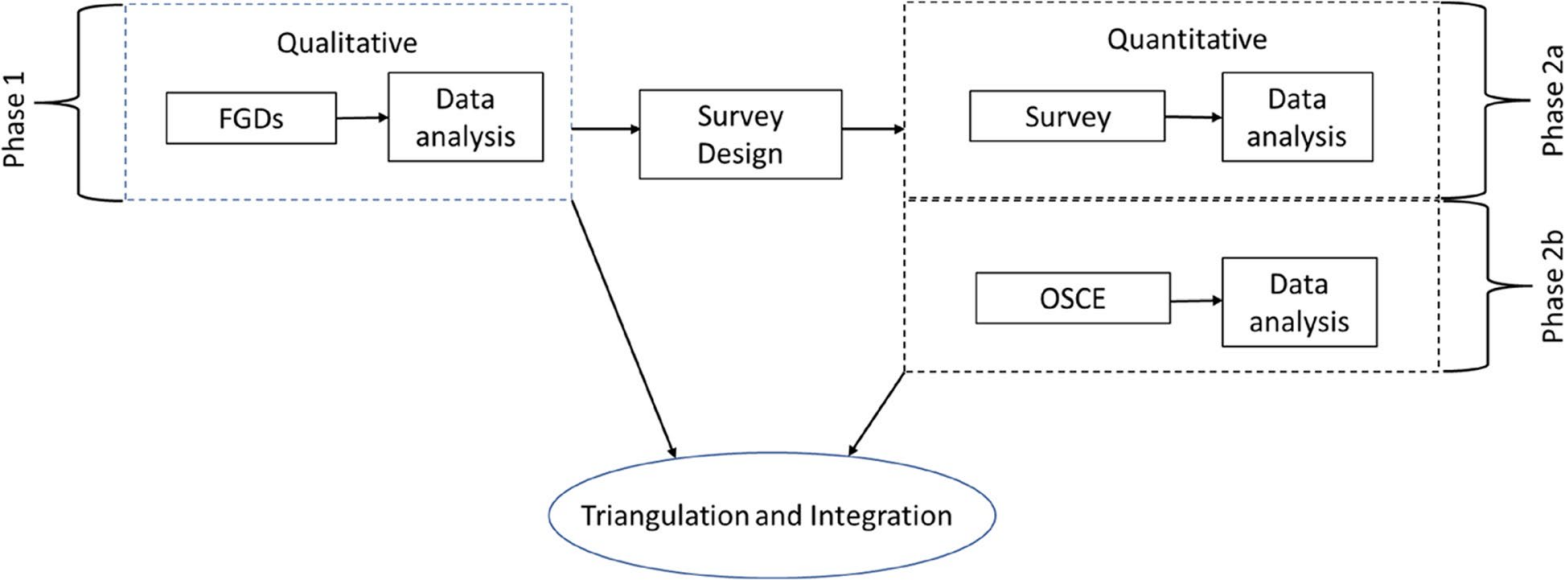
Flow diagram showing exploratory mixed methods design. In Phase 1 (qualitative strand) transcripts from focus group discussions (FGDs) were analysed and used to inform the design of the survey questions used in the first part of the quantitative strand (Phase 2a). In Phase 2b, performance scores of the disrupted cohort on the Objective Structured Clinical Examination (OSCE) were compared with that of previous cohorts

This study employed an exploratory sequential mixed methods design [9] of two phases, which included an initial qualitative phase (phase 1) of data collection and analysis (focus group discussions), followed by a phase of quantitative data collection (phase 2a) and analysis (surveys). During phase 2b, average 2020 student cohort performance in OSCE stations was compared to that attained by previous cohorts in the identical station offered in a pre-COVID examination (either 2018 or 2019). Results from both phases of the study were triangulated and integrated to allow richer interpretation of the phenomena in line with the study aims (Fig. 1).

Demographic characteristics have been implicated as contributing to differences in OSCE performances [10, 11], thus simple demographic descriptors of the three student cohorts (2018, 2019, 2020) were also examined to discern any significant variability that may provide alternative explanation for any differences in OSCE performance found. Informed consent was obtained from all focus group discussion (FGD) participants, who were provided with a written participant information sheet and provided researcher contact details (CJ) for further queries. Verbal consent was obtained and recorded at the commencement of the FGDs. Based on the analysis of the FGDs, a survey was designed and administered to whole of cohort after the end of year OSCE examination (Table 2). Anonymized whole of cohort data was provided to the research team for demographic and assessment outcome analysis, ensuring that no individual was identifiable. This process was in accordance with that approved by the University of Notre Dame Australia’s Human Research Ethics Committee approval (ref 2020-142S).

**Table 2:** Timeline of study phase 1 and 2a activities

Date	Activity
21 September	Digital invitation to participate
24 September	Digital reminder to participate
28 September	Digital reminder to participate
7 October (am group)	FGD—group 1
7 October (pm group)	FGD—group 2
9 October (am group)	FGD—group 3
October–November	Transcription and coding
30 November	Online survey

### Phase 1

#### Focus group discussions

Focus group discussions (FGDs) using purposive sampling were used to gather qualitative data concerning Year 2 students’ perceptions regarding their clinical skills learning during 2020. Focus group discussions were used to extrapolate an enriched exploration of ideas in relation to the impact of clinical skills teaching delivery disruptions by allowing interaction of participants [12].

FGD questions (Appendix 1) were informed by the results of a literature search on student perceptions of online learning, and early pandemic works on student perceptions of the learning impacts of lockdowns and teaching changes. Questions were further refined based on discussions with experts at national clinical assessment benchmarking collaboration (Australian Collaboration for Clinical Assessment in Medicine) meetings, and further discussions within the research team, which included the student researcher perspective.

The FGD questions were framed according to two broad areas: (i) student perceptions regarding clinical skills learning that was most impacted by the transition to online teaching during peak COVID-19 pandemic conditions in 2020; and (ii) student perceptions regarding the impact on clinical skills learning following the transition back to face-to-face clinical skills teaching once physical distancing regulations provided for this.

The FGDs occurred in October 2020, prior to the Year 2 summative examinations (held on campus in November). Although limited face-to-face teaching had resumed during the second half of 2020, the researchers chose to conduct the FGDs online. This decision was made in the context of a tenuous COVID environment, where meeting on campus was largely restricted to students scheduled for practical clinical skills learning in order to protect students and staff from COVID-19 infection. The researchers felt that the students were familiar with meeting virtually to share ideas and discussions during tutorials, and therefore conducting the FGDs online was a reasonable compromise during the pandemic. One of the researchers (CJ) who is not part of the teaching team for this cohort of students interviewed the participants to reduce potential power differentials between the interviewer and interviewees, as well as to encourage collaborative discussions amongst the participants [13].

##### Population and recruitment

Participants were recruited from the Year 2 student cohort (of 120 students) at the University of Notre Dame, School of Medicine, Sydney (SoMS), with invitations being distributed via email (using student university addresses) and via the Year 2 student Facebook page. Three separate invitations were sent to encourage participant involvement. Each invitation outlined the study objectives and provided information about how to register for participation.

##### Focus group discussion format

FGDs were conducted using the Zoom video conferencing platform, with participants’ videos and microphones remaining on at all times. Three separate focus groups were held, with 7 students (plus the interviewer) in each group. Each FGD ran until data saturation was complete, at an average of 36 min per FGD. Discussions were recorded using the Zoom software’s internal recording feature, with the recorded verbal consent of participants.

##### Analysis

Audio files were de-identified and transcribed via an external transcription service. Thematic analysis [14] of the transcripts was undertaken by CJ, with responses being broadly classified according to both deductive coding according to the research question, with further inductive review for emergent themes. Final consensus regarding the coding was settled by iterative discussions involving the research team (CR, SS, CJ). The identified themes are presented using illustrative quotes that are affixed with the individual participant number (P1-7) and the focus group they attended (FG 1–3). Identified themes informed the basis of the questions developed for the online survey.

### Phase 2a

#### Online survey

A follow-up online survey of 12 Likert-scale response format questions was developed from the thematic analysis of the FGD responses. The survey (Appendix 2) aimed to investigate the prevalence of the experiences described in the themes derived from the FGDs. This survey was distributed to the whole Year 2 cohort, to gather quantitative data from students regarding their experiences and perceptions of online clinical skills learning, and the impact of disrupted teaching delivery during 2020.

The survey was distributed to the Year 2 cohort following the Year 2 summative OSCE examination in November 2020, but before assessment results were returned.

##### Population and recruitment

All Year 2 students were invited to participate in the survey, and invitations were distributed to the whole year cohort via email using student university addresses, as well as the Year 2 student Facebook page.

##### Analysis

Descriptive statistics were used to determine percentages, means and standard deviations of the online survey responses.

### Phase 2b

#### Population

This phase examined whole of cohort, deidentified clinical performance and demography data of the Year 2 students at SoMS.

#### OSCE assessment

The 2020 Year 2 OSCE consisted of 8 stations run over 2 sites on a single day, according to best practice principles of standardisation of examination conditions, assessors, and simulated patients [15]. The team who blueprinted, standardised, and ran the OSCE had significant experience and expertise in OSCE processes. The same team had blueprinted and run the OSCEs for the 2018 and 2019 cohorts.

The OSCE stations were authored in alignment with the curriculum, then circulated for review and comment by clinical tutors with subject matter expertise. Final revisions were then made, ensuring content validity of the stations.

Pearson’s correlation coefficient (*r*) was calculated to measure the correlation of station scores with overall OSCE performance as a measure of internal consistency (construct validity) of the examination. All stations had similar *r* values pre- and post-COVID, had positive *r* values, and were deemed to have acceptable reliability.

#### Evaluation of the impact on student OSCE performance

To determine whether teaching disruptions impacted student clinical performance, identical stations included in the 2020 Objective Structured Clinical Exam (OSCE) were matched to a previous offering (either in the 2018 cohort or 2019 cohort). Student performance on each of these matched stations was compared using a Student’s independent t-test (α < 0.05). Where there was a significant disparity in the marks returned by multiple examiners for a single student in performing a particular station, that result was excluded from the study. Only consistent, reliable station scores were included in this comparative study.

#### Evaluation of the demography of the student cohorts

An analysis (using the chi-square test and ANOVA) was also undertaken to compare the demography of the student cohorts in 2018, 2019, and 2020, with a view to determining whether these cohorts were comparable with respect to: gender composition, category of previous degree (Health Professions, Sciences, or Other), and age in years when they completed the OSCE.

## Results

### Phase 1 results

#### Focus group discussions

**Fig. 2:**
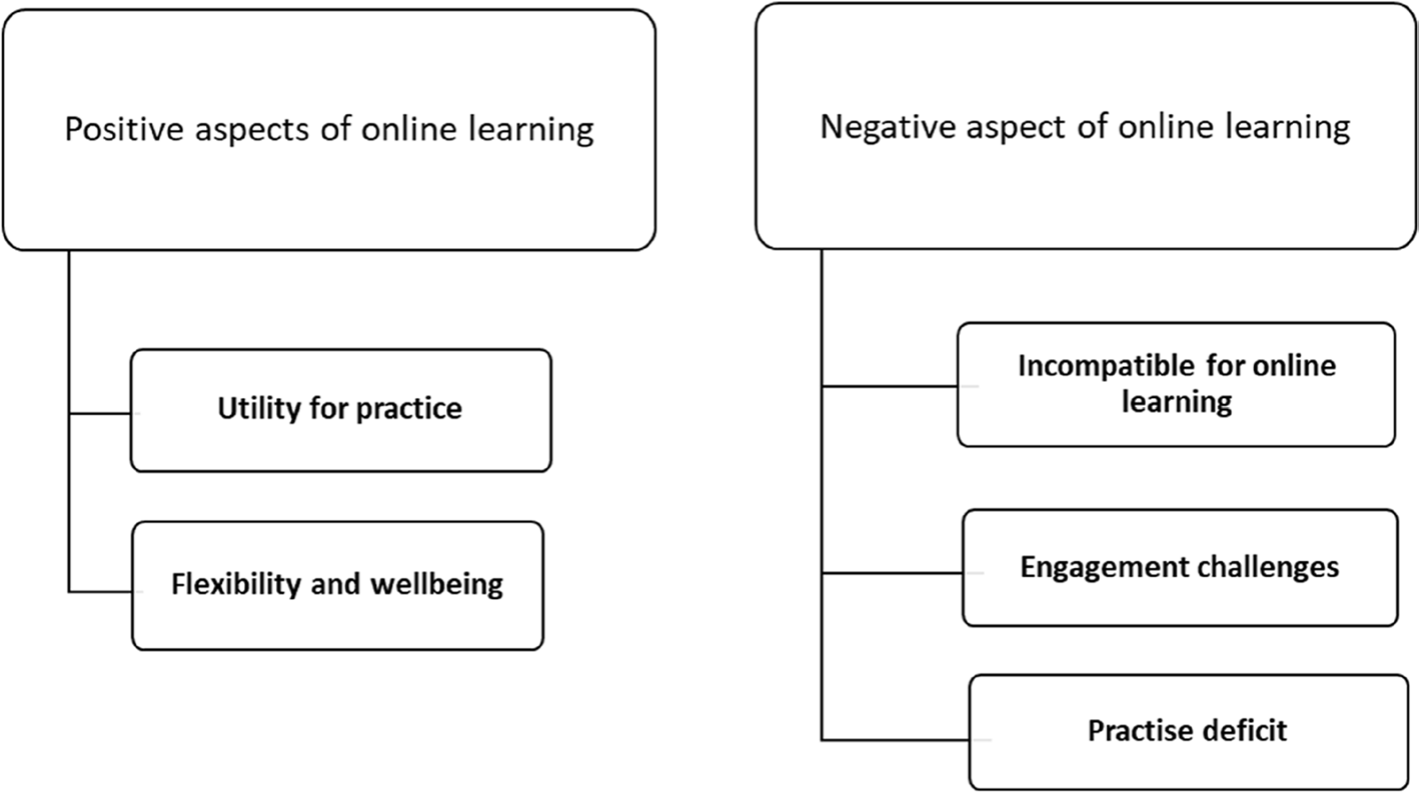
Major themes derived from the analysis of the focus group discussions

Twenty-one students participated in the FGDs; 13 female and 8 male students, comprising 18% (21/120) of the cohort. Data saturation occurred in the third focus group, where recurrence of major themes occurred without the emergence of new themes. Further sampling seeking additional themes was not conducted due to feasibility limitations: we received no further responses from willing volunteer participants. Thematic analysis identified the following themes: broad benefits of online learning; and negative impacts (Fig. 2).

### Benefits of online learning

#### Theme 1: utility for practice

Students felt that selecting the correct skills content for online sessions was important for minimising a negative impact on their learning.

*Doing histories was quite good I suppose, you can really punch through them and you could work around them fairly easily. *(P4 FG1)

Of interest, students noted that practising these skills via an online format had potential utility in preparing students for future Telehealth practice:

*If the right skills are taught online such as investigations (ECGs for example) and history-taking which can be done in the Telehealth format, I think those skills can be developed online. *(P3 FG3)

The extra time spent focusing on learning clinical reasoning skills (such as investigations interpretation and diagnostic formulation) during online sessions was perceived as beneficial for student learning of clinical reasoning skills.

*I personally think my clinical reasoning has really benefited from [online learning]. Because I haven't been focusing on the technical stuff and the skillsets and I've been focusing on building frameworks and differentials.* (P2 FG2)

### Theme 2: flexibility and wellbeing

It was identified that shifting to an online format for clinical skills learning conferred personal and social benefits for students who were experiencing the hardships of the pandemic lockdowns.

*I've noticed I've got more hours in the day so I'm not travelling an hour and a half both ways… And also, just whenever we clock out for a lunch break it’s nice to be in your own house, you can chuck on Netflix or talk to your partner and I enjoy that.* (P7 FG2)

*The transition to online learning was a benefit for me because that meant I could go home to my family. It was good for my wellbeing so that was an advantage.* (P2 FG3)

*It was really good to be able to be with our supports and go home. So that really was beneficial to me in the context of the pandemic, not just because it was online.* (P7 FG3)

### Negative impacts

#### Theme 3: incompatible for online learning

In contrast, students felt that learning ‘hands on’ clinical skills was negatively impacted by the transition to online learning during the 2020 COVID outbreak. To this end, basic procedural skills (such as cannulation and venepuncture) and physical examination skills were perceived as being the most difficult to learn via an online platform:

*I'd say is that I think, overall, learning skills online wasn’t very productive in some areas, particularly examinations and procedures.* (P2 FG3)

*So, for example, until I touched those venepuncture or cannulation kind of models I wasn’t sure the angle or the pressure and it’s like, that kind of small coordination that you really need to do it in person rather than online.* (P1 FG2)

#### Theme 4: engagement challenges

In addition to the difficulty of practising ‘hands on’ clinical skills via an online format, the virtual environment could limit engagement in learning.

*When you're on Zoom, your recall and ability to absorb the information sort of reaches a threshold, and then you're done. And I think that threshold comes much faster online than it does in person.* (P6 FG3)

Furthermore, where unstable internet access was experienced, students reported additional irremediable disruption to learning.

*When COVID first started I had the most dismal internet. So I think the technical difficulties were a challenge at times.* (P3 FG2)

#### Theme 5: practise deficit

Moreover, students felt there were ongoing negative effects impacting their clinical skills acquisition when they transitioned back to face-to-face learning. Of note, students felt that ongoing physical distancing regulations (and concomitant lack of room availability) following a return to campus meant that there were inadequate opportunities for students to truly catch-up on missed skills practise following the recent period of online learning:

*So, the availability of space to practice and practising with peers has been very limited this year. So, I personally would rate my development of skills to be very, very minimal.* (P3 FG3)

*So, this year, not having that interaction with other students, just means that I haven't got nearly as much practice with my clinical skills. I think that this year there's a big gulf in my skills.* (P4 FG1)

### Phase 2a results

#### Online survey

Twenty-seven students responded to the follow-up online survey, representing a 22.5% response rate.

The survey results showed 85% of respondents agreed that online learning helped to prepare them for delivering Telehealth in the future, whereas time effectiveness was experienced as a benefit by only 33% of respondents. Additionally, students either agreed or strongly agreed that Zoom fatigue (78%) and technical issues (52%) had negative effects when learning clinical skills online (Table 3).

**Table 3:** Survey results showing perceived benefits and disadvantages of online learning of Clinical Skills, *n* = 27

	**Agree (%)**	**Neutral (%)**	**Disagree (%)**
**Benefits**			
Telehealth training	85	11	4
Time effective	33	7	59
**Disadvantages**			
Decreased concentration span	80	8	12
Technical difficulties	52	12	35

**Fig. 3:**
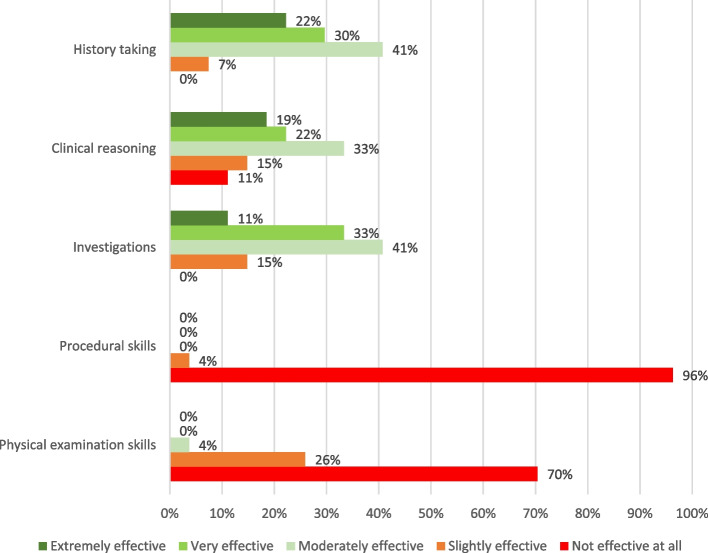
Perceived effectiveness of online teaching of specific clinical skills by Year 2 medical students

Survey results indicated that online learning was found moderately to extremely effective for teaching history-taking skills (93%), interpreting investigations (85%) and other clinical reasoning skills (such as diagnostic reasoning) (74%), though procedural and physical examination skills were unsuited to online learning (100% and 96% disagreement respectively) (Fig. 3).

**Fig. 4:**
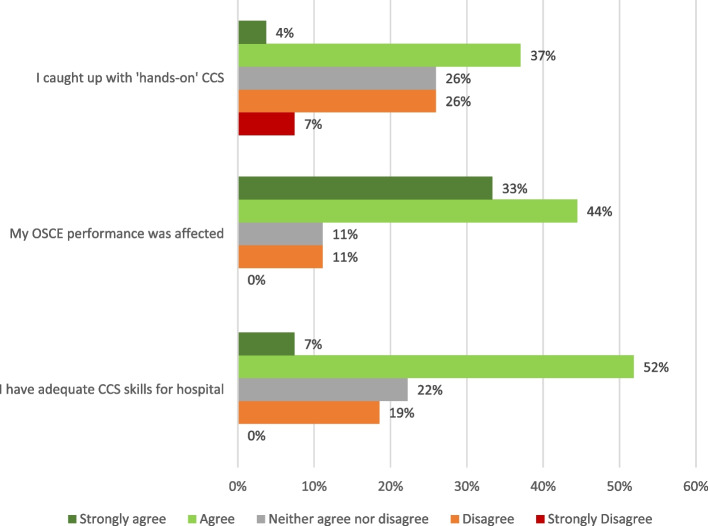
Perceived effect of transitioning back to face-to-face teaching by Year 2 medical students

Regarding the impact of shifting back to face-to-face teaching, survey results showed 41% of respondents felt they caught up with clinical skills learning, though 77% agreed that their end-of-year summative clinical examination performance was affected by clinical skills teaching disruptions during 2020. More than half (59%) of the surveyed students agreed that their skills were adequate for the transition to clinical placements in Year 3 (Fig. 4).

### Phase 2b results

#### OSCE performance on history-taking stations

**Fig. 5:**
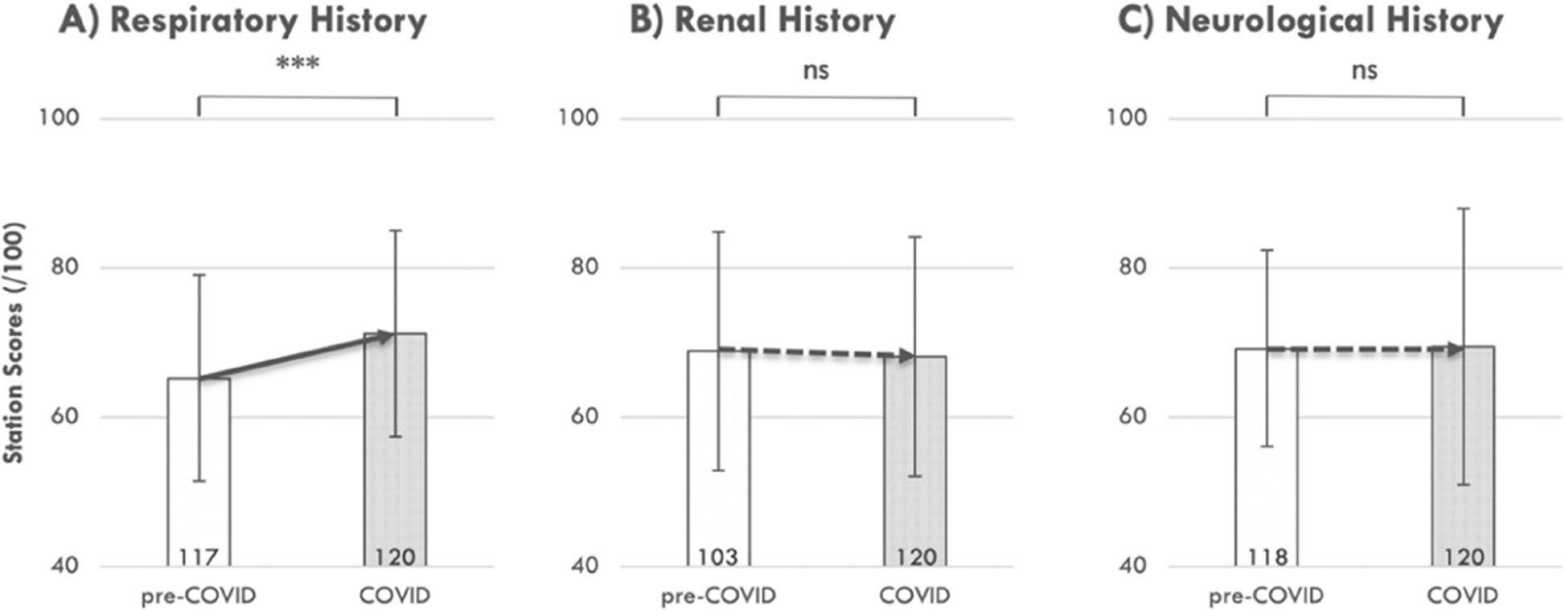
OSCE performance in respiratory history-taking (**A**) was significantly improved in the COVID-impacted cohort, but other history-taking stations showed no change (**B** & **C**). OSCE performance was compared in matched history-taking stations between the 2020 (COVID-impacted) and either the 2018 (**A**) or 2019 (**B** and **C**; pre-COVID) examination. Statistical comparisons were made using independent t-tests; ***, *p* = 0.001 (significant), ns, non-significant. The number of individual students in each cohort is provided in the base of each bar

Three history-taking stations were compared between the COVID-impacted (2020) and pre-COVID: either 2018 (n = 118) or 2019 (n = 117) second-year student cohorts in this study, namely: Respiratory, Renal, and Neurological. The 120 students in the COVID-impacted year demonstrated significantly higher station scores on the Respiratory History (M = 71%, SD = 14) compared to the 117 students in the pre-COVID year (2019; M = 65%, SD = 14), t(235) = -3.33, *p* = 0.001 (***); (see Fig. 5.A). However, no significant differences were observed between the COVID and pre-COVID student performance on the Renal History station, nor Neurological History station (see Fig. 5.B and C, respectively).

### OSCE performance on physical examination stations

**Fig. 6:**
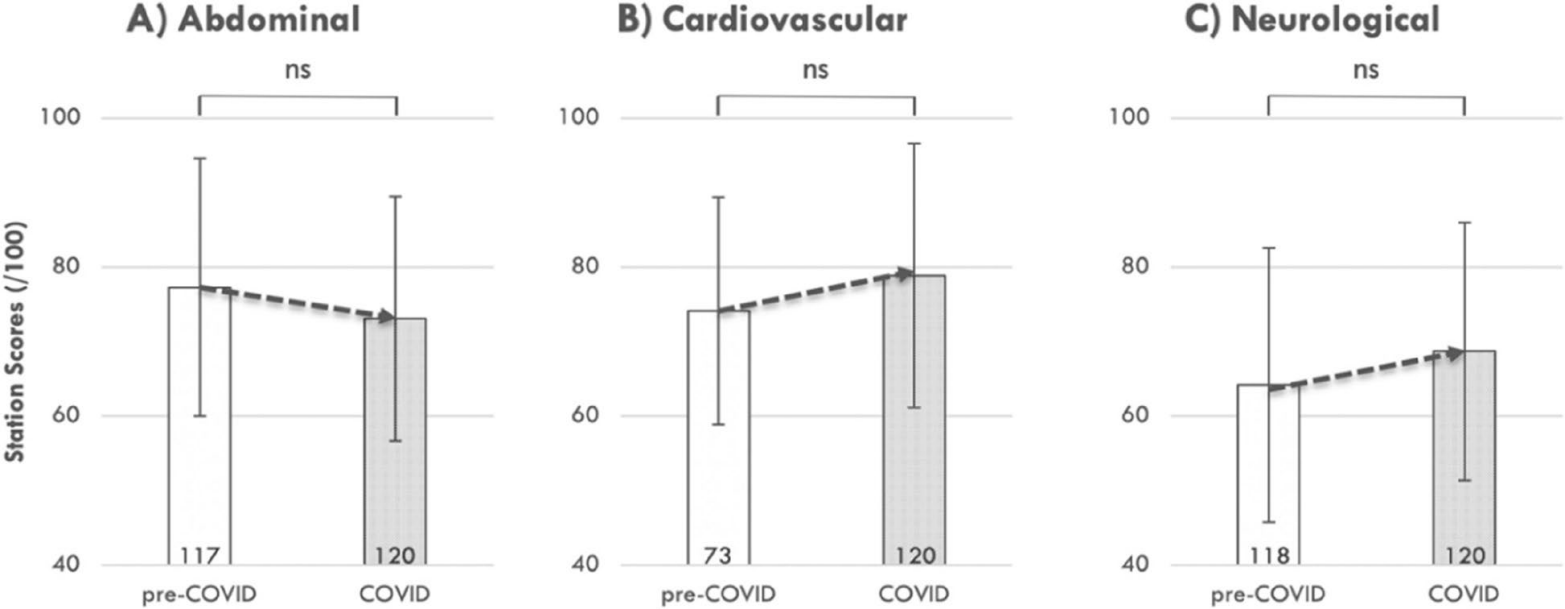
OSCE performance in physical examination in the abdominal (**A**), cardiovascular (**B**), and neurological (**C**) systems showed no significant changes between the COVID-impacted and pre-COVID cohorts. OSCE performance was compared in matched physical examination stations between the 2020 (COVID-impacted) and either the 2018 (**C**) or 2019 (**A** and **B**; pre-COVID) examination. Statistical comparisons were made using independent t-tests; ns, non-significant. The number of individual students in each cohort is provided in the base of each bar

Three physical examination stations were compared between the COVID-impacted (2020) and pre-COVID (either 2018 or 2019) students, namely: Abdominal, Cardiovascular, and Neurological. The apparent differences observed between the COVID and pre-COVID student performance on all of the physical examination stations in this study were not significant (see Fig. 6).

#### OSCE performance on procedural skill and clinical reasoning stations

Two other stations were compared between the COVID-impacted (2020) and pre-COVID (either 2018 or 2019) students, namely: Abdominal Investigations and Venepuncture. The Abdominal Investigations station measures student performance in interpreting blood test and radiological results with a focus on clinical reasoning skills.

**Fig. 7:**
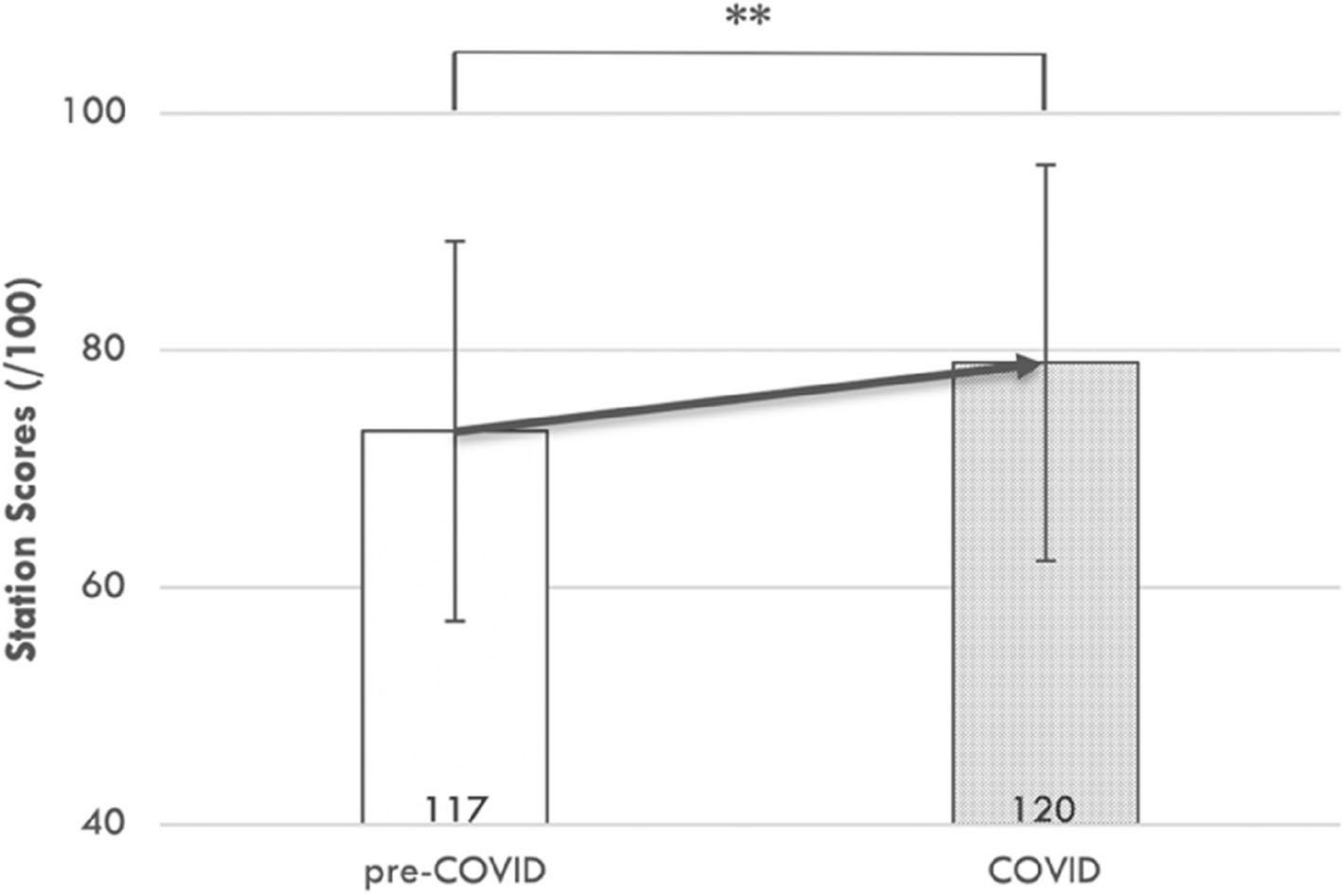
OSCE performance in clinical reasoning, demonstrated in the abdominal investigations station, was significantly improved in the COVID-impacted cohort. OSCE performance was compared in a matched abdominal investigations station between the 2020 (COVID-impacted) and the 2019 (pre-COVID) examination. This statistical comparison was made using an independent t-test; **, *p* < 0.01 (significant). The number of individual students in each cohort is provided in the base of each bar

The 120 students in the COVID-disrupted year demonstrated significantly higher station scores on Abdominal Investigations (M = 79, SD = 17) compared to the 117 students in the pre-COVID year (2019; M = 73, SD = 16), t(235) = -2.72, p = 0.007 (**); see Fig. 7.

**Fig. 8:**
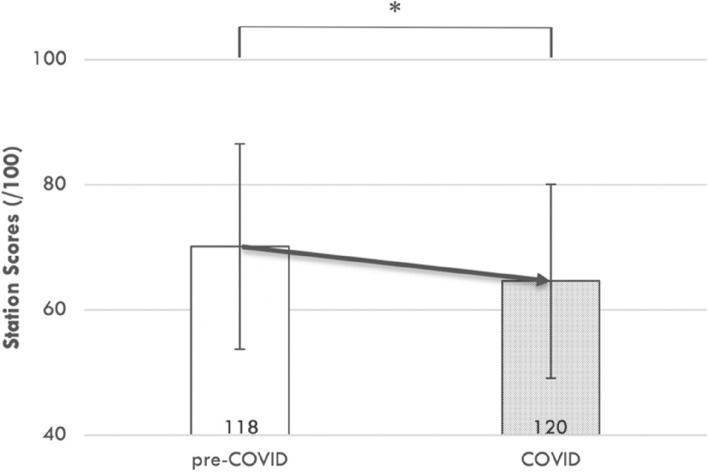
OSCE performance in procedural skills, demonstrated in the venepuncture station, was significantly worse in the COVID-impacted cohort. OSCE performance was compared in a matched venepuncture station between the 2020 (COVID-impacted) and the 2018 (pre-COVID) examination. This statistical comparison was made using an independent t-test; *, *p* < 0.05 (significant). The number of individual students in each cohort is provided in the base of each bar

The Venepuncture station measures student performance in collecting ‘blood’ from a vein using a fluid-filled model. This was the only station considered in this study where the 120 students in the COVID-disrupted year (M = 65, SD = 16) demonstrated significantly lower station scores compared to the 118 students in the pre-COVID year (2018; M = 70, SD = 16), t(236) = 2.38, *p* = 0.02 (*); see Fig. 8. It is noteworthy that access to physical models to practice Venepuncture technique were markedly limited due to prevailing COVID restrictions in the period leading up to the OSCE.

#### Demography of student cohorts

A chi-square test of independence was performed to compare the distribution of gender (Male, Female, or No Response) between the 2018 (pre-COVID), 2019 (pre-COVID) and 2020 (COVID) second-year student cohorts. There was no significant variation in the distribution of gender associated with cohort,*X*^2^ (4, *N* = 355) = 7.34, *p* = 0.12 (ns). Similar analysis was undertaken to compare the distribution of category of previous degree (Health Professions, Sciences, Other, or No Response). As with gender, there was no significant variation in the distribution of category of previous degree associated with cohort, *X*^2^ (6, *N* = 355) = 2.53, *p* = 0.87 (ns). Finally, a one-way between subjects ANOVA was conducted to compare the distribution of age in years between the 2018 (pre-COVID), 2019 (pre-COVID) and 2020 (COVID) second-year student cohorts. There was no significant variation in age in years at the for the three cohorts [F(2, 352) = 1.76, *p* = 0.17 (ns)]. Taken together, these results show that there were no significant differences between the two pre-COVID (2018 and 2019) student cohorts and the COVID-impacted cohort (2020) with respect to gender, category of previous degree, nor age in years when they completed the OSCE.

## Discussion

Whilst there have been large international surveys of student perceptions of adaptations to medical education during the COVID-19 pandemic [16, 17], this study allows the juxtaposition of student perceptions of learning impact due to teaching disruptions with their measured skills performance compared to previous undisrupted cohorts.

Specifically, this begins to address the call for a drilling down into the impact of specific educational interventions enacted in response to the pandemic on target populations using an objective outcome measure [17, 18].

In this study, participating disrupted students reported perceiving an impact on their OSCE performance, and a preference for face-to-face teaching for physical examination and basic procedural skills sessions. However, on objective measurement, the cohort performed at least as well (if not better) on all OSCE stations, apart from basic procedural skills, as previous cohorts. This overall performance aligns with the cohort reporting that, despite disrupted learning, they felt adequately prepared to commence clinical rotations in the coming year.

Students felt that, from their experience, history-taking and clinical reasoning skills are amenable to synchronous online learning. Aligned with this perception, when comparing OSCE performance results of COVID-disrupted (2020) students with pre-COVID (2018 and 2019) students, analyses showed that COVID-impacted students performed at a comparable level on identical history-taking and clinical reasoning OSCE stations as their pre-COVID colleagues. This highlights that online teaching was an effective modality for students to develop history-taking skills, and aligns with the findings of other medical schools [18].

The fact that COVID-impacted students performed better at history-taking involving the respiratory system than pre-COVID students may reflect that there was a greater societal exposure and personal emphasis on learning this specific skill during a pandemic year. Similarly, the disrupted cohort’s superior performance in the clinical reasoning station may indicate an educational benefit from increased time and focus spent on that activity due to the hiatus from physical examination skills teaching.

In the model of disruption experienced by participating students, physical examination was taught online as an abbreviated discussion of technique and approach, supported by audiovisual resources, followed by face-to-face catch-up practical sessions when pandemic social distancing restriction eased. This model of disruption resulted in non-inferior skills acquisition as measured on objective testing, which aligns with previous findings regarding a similar “flipped classroom” style of online physical examination skills teaching [19].

The reported student perception that procedural skills were not able to be effectively taught online aligns with international survey results [16]. Specific to our cohort, despite practical face-to-face catch-up sessions, the disrupted student group performed significantly poorer at the practical procedural skills (venepuncture) than their pre-COVID colleagues. Our qualitative findings indicate that students lacked opportunities to access on-campus simulation equipment necessary to practise the skill, which strongly suggests schools will need to plan for either creating covid-safe practise opportunities with simulation equipment, or future remediation training of cohorts that have experienced exclusion from campus facilities.

Participants reported additional benefits to online synchronous distance education, including time efficiency, personal convenience and increased learning regarding telehealth, which aligns with the findings of other surveys [16, 18]. Our students also reported connectivity issues, domestic interruptions and “Zoom fatigue” as negative aspects of their online learning experiences, again in alignment with international surveys [16, 18].

In this study, demographic analyses show that there is no significant difference between the 2018, 2019 and 2020 Year 2 student cohorts with respect to gender, age and category of previous degree, highlighting that these factors have not impacted the actual outcomes of data analyses, suggesting that the findings are in fact most likely to be due to whether the academic year was COVID-disrupted or not.

Synchronous distance education has high acceptability for health professions education [20], and has proven non-inferiority (and possible benefit) on measured assessment outcomes when employed during knowledge or reasoning educational activities [21, 22]. This study furthers these findings in suggesting that some practical skills are amenable to online learning (history-taking and clinal reasoning), while a blended asynchronous delivery can be successful for others (physical examination skills). Though limited to a single practical skill, our findings suggest schools will need to allow adequate access to simulation facilities for students to acquire basic procedural skills e.g., venepuncture that may have been compromised due to pandemic disruptions to teaching. The approach taken in this study, where an objective measure is used to compare skills acquisition during disruptions with previous cohorts, has the benefit of highlighting likely deficits to inform the design of targeted remediation activities.

### Limitations

This study comprises participants from a single medical school site, which decreases the generalizability to other contexts and health professions. It is noted that conducting the FGDs online could have hindered the sharing of ideas and perspectives during discussions. However, the familiarity of students in meeting online is likely to have minimised this impact. While this is a cohort study, where differences observed in performance may be attributable to cohort differences, there has been no distinct demographic differences shown on cohort comparison which could provide an alternative explanation for our results.

The low response rate to the online survey reduces the reliability of the phase 2a findings. The online survey was administered after the Year 2 summative OSCE examination in November 2020, but before the students received their assessment results. The low response rate to the survey may have been impacted by the timing of its administration, noting that student motivation to participate in academic events may be at a low point once assessments have been completed for the year. However, the high concordance of our findings with larger international survey findings (as outlined above), increases the confidence that we are reporting accurate phenomena.

In the future, it is anticipated that medical schools and student groups will have greater experience with, and more thorough design of, online learning which will decrease the durability of the findings of this study.

Future studies may involve multiple institutions or benchmarking consortia investigating if pandemic disruptions result in measurable and meaningful differences in skills learning compared to previous cohorts. It would also be of interest to examine if measured differences persisted longitudinally, or if they were “caught up” during subsequent years of training.

## Conclusion

Our findings concur with prior studies that suggest COVID teaching disruptions impact medical student confidence in their clinical skills learning. Medical schools enacting rapid adaptations to skills learning in response to pandemic public health directives will need to be mindful to proactively address this psychological repercussion.

In our blended design, the rapid re-introduction of face-to-face skills intensives resulted in non-inferior measured performance compared to previous cohorts, except in basic procedural skills, which likely require more rehearsal opportunities with simulation equipment. Reassuringly, clinical reasoning and history taking skills are amenable to synchronous online learning. The findings of this study add to the literature that can be referenced when future-proofing clinical skills training in response to pandemics or other unfortunate catastrophic disruptions such as those caused by climate change.

## Supplementary Information

**Additional file 1.** 

**Additional file 2.** 

## Data Availability

The datasets used and/or analysed during the qualitative phase of the current study are available from the corresponding author on reasonable request. Demographic and assessment outcome data requests would be forwarded by the corresponding author to the relevant Human Research Ethics Committee for consideration.

## Abbreviations

OSCE: Objective Structured Clinical Exam
COVID: Coronavirus disease
M: Mean (or average) of the sample
SD: Standard deviation of the sample
t: *t*-Statistic value associated with a Student’s t-test
p: Probability associated with statistic
ns: Non-significant, p > = α, accept the null (no change) hypothesis
*X*^2^: Chi-squared value associated with a Chi-squared test
*N*: Number (or count) of the sample
ANOVA: Analysis of variance
F: *F*-Statistic value associated with an ANOVA
N.B.: different journals will have different guidelines related to how abbreviations are to be used and presented. Many journals will provide a list of common abbreviations that do not require explanation, which may include terms like mean, standard deviation and ANOVA
