# Supplementary material for: The impact of pandemic disruptions on clinical skills learning for pre-clinical medical students: implications for future educational designs
Source: BMC Med Educ. 2023 May 23;23:364. doi: 10.1186/s12909-023-04351-9 (PMC10202529; doi:10.1186/s12909-023-04351-9)
Supplement: Supplementary file 1 — Additional file 1. [file 12909_2023_4351_MOESM1_ESM.docx]

**Appendix 1**

**Focus Group Discussion (FGD) prompting questions**

**FGD questions**

1. Can you describe your experience of learning clinical skills online?
2. Can you describe any advantages (or new opportunities) to learning clinical skills (such as history-taking, physical examinations and other clinical skills) online?
3. Can you describe any disadvantages (or missed opportunities) to learning clinical skills (such as history-taking, physical examinations and other clinical skills) online?
4. Are there any particular clinical skills that were affected more than others when learning online? In what way(s)?
5. What do you think could have been done to make your learning of clinical skills online more effective?
6. What was your experience of involving volunteer simulated patients to practise clinical skills via an online delivery mode?

**FGD prompting questions to facilitate discussions**

1. Has anybody else experienced something similar?
2. Does anybody else have an additional comment to add here?
3. Has anybody experienced something different to that?
