# Supplementary material for: The impact of pandemic disruptions on clinical skills learning for pre-clinical medical students: implications for future educational designs
Source: BMC Med Educ. 2023 May 23;23:364. doi: 10.1186/s12909-023-04351-9 (PMC10202529; doi:10.1186/s12909-023-04351-9)
Supplement: Supplementary file 2 — Additional file 2. [file 12909_2023_4351_MOESM2_ESM.docx]

Appendix 2:

**Online survey**

1. There were a number of expected and unexpected benefits of learning clinical and communication skills via an online platform. Indicate to what extent you agree with the following:

|  | Strongly disagree | Disagree | Neither agree nor disagree | Agree | Strongly Agree |
| --- | --- | --- | --- | --- | --- |
| 1. Learning via an online platform exposed me to some of the issues I might encounter in the future when delivering Telehealth |  |  |  |  |  |
| 1. Learning via an online platform was more time effective than learning face-to-face |  |  |  |  |  |

1. There were a number of expected and unexpected disadvantages of learning clinical and communication skills via an online platform. Indicate to what extent you agree with the following:

|  | Strongly disagree | Disagree | Neither agree nor disagree | Agree | Strongly Agree |
| --- | --- | --- | --- | --- | --- |
| 1. My learning via an online platform was negatively impacted by Zoom fatigue |  |  |  |  |  |
| 1. My larning via an online platform was negatively impacted by computer and wifi accessibility and stability issues |  |  |  |  |  |

1. To what extent do you agree with the following statement: Online delivery of teaching was effective when learning the skills of….

|  | Strongly disagree | Disagree | Neither agree nor disagree | Agree | Strongly Agree |
| --- | --- | --- | --- | --- | --- |
| 1. History-taking |  |  |  |  |  |
| 1. Physical examinations |  |  |  |  |  |
| 1. Procedural skills (e.g cannulation, lumbar puncture) |  |  |  |  |  |
| 1. Ordering and interpreting investigations (e.g ABG, Xray, ECG) |  |  |  |  |  |
| 1. Demonstration of clinical reasoning |  |  |  |  |  |

1. During Semester 2 of 2020, clinical and communications skills teaching re-transitioned to a face-to-face format. To what extent do you agree with the following:

|  | Strongly disagree | Disagree | Neither agree nor disagree | Agree | Strongly Agree |
| --- | --- | --- | --- | --- | --- |
| 1. I was able to catch up on my “hands on” skills learning following a transition back to face-to-face teaching |  |  |  |  |  |
| 1. I feel that my performance at the Year 2 summative OSCE was impacted by a temporary transition to online learning in 2020 |  |  |  |  |  |
| 1. I feel confident that I have developed adequate clinical skills to progress to the next year evel |  |  |  |  |  |
